# Changes in Nurse-Patient Communication Through Health Technologies and Nursing Practices to Recognize and Support Limited Digital Health Literacy: Qualitative Study

**DOI:** 10.2196/82272

**Published:** 2026-03-26

**Authors:** Eline Mariosé Dijkman, Jobke Wentzel, David Edvardsson, Carine Doggen, Constance Helène Christine Drossaert

**Affiliations:** 1 Department of Surgery, Isala Hospital, Zwolle, The Netherlands; 2 Health Technology & Services Research, University of Twente, Enschede, The Netherlands; 3 Department of Health and Social Studies, Windesheim University of Applied Sciences, Zwolle, The Netherlands; 4 Department of Nursing, Swinburne University of Technology, Melbourne, Victoria, Australia; 5 Sahlgrenska Academy, University of Gothenburg, Gothenburg, Sweden; 6 Clinical Research Centre, Rijnstate Hospital, Arnhem, The Netherlands; 7 Department of Psychology, University of Twente, Drienerlolaan 5, Enschede, 7522 NB, The Netherlands, 31 053 489 911

**Keywords:** health literacy, digital health literacy, nurse-patient communication, hospitals, tailored communication, eHealth literacy, nursing, hospital

## Abstract

**Background:**

In the past decade, the use of health technologies, such as telemonitoring, video consultations, and patient portals, has increased. However, it remains unclear how these technologies have influenced nurse-patient communication. Additionally, little is known about the role nurses play in recognizing and supporting limited (digital) health literacy patients.

**Objective:**

This study aimed to explore which health technologies are currently being used in a hospital context and how nurse-patient communication has changed as a result. Furthermore, we sought to identify the practices nurses use and the barriers they experience in recognizing and supporting patients with limited digital health literacy.

**Methods:**

This is a qualitative descriptive study that used semistructured interviews with nurses working in a hospital (n=21). The interview guide was partly based on the 6-function model of medical communication by de Haes and Bensing. All interview transcripts were analyzed by 2 independent coders using a combination of deductive and inductive approaches.

**Results:**

According to the nurses, health technologies have impacted all 6 functions of nurse-patient communication. They noted improvements in gathering information, providing information, enabling disease and treatment management, and responding to patients’ emotions. In contrast, technology made fostering the relationship more difficult, and technologies were seldom used in shared decision-making. Nurses identified limited digital health literacy through intuition, observation of verbal and nonverbal cues, and direct questioning. To support patients with limited digital health literacy, nurses relied on building trust, involving the social network, tailoring communication, and offering additional support. High workload and limited knowledge were the main barriers to applying these practices.

**Conclusions:**

Our findings show that health technologies have significantly influenced nurse-patient communication in the hospital setting. The results highlight the need for tailored training programs to strengthen nurses’ competencies in identifying and supporting patients with limited digital health literacy. This is essential to ensure more comprehensible and accessible care and promote equitable patient engagement with health technologies.

## Introduction

One of the essential elements of nursing care is communication with patients and their relatives [1]. Communication between nurses and patients serves different functions. The 6-function model of de Haes and Bensing [2] distinguishes the following functions from the viewpoint of the care professional: (1) fostering the relationship, (2) gathering information, (3) providing information, (4) decision-making, (5) enabling disease and treatment-related behavior, and (6) responding to the emotions of patients. In practice, these functions overlap and interact during nurse-patient communication. Nevertheless, they are important for positive patient outcomes, including patient satisfaction, adequate diagnosis and treatment, treatment adherence, and even health [3,4].

Over the past decade, the rapid rise of the internet and digital health technologies has begun to reshape the context in which these communication functions take place. Nowadays, patients have access to health information via the internet and social media and to specific information about their own health status and treatment via the hospitals’ patient portal. Apps exist for self-management, and the telemonitoring of vital functions may provide care professionals with continuous insight into bodily functions, which was formerly not possible. In addition, nursing consultations are increasingly being conducted digitally, via email, chat, or video conferencing, and health education is frequently supported by websites and videos.

These developments offer various benefits, such as improved patient insight into their health and reduced care costs and patient burden due to fewer hospital visits [5]. However, the benefits may not exist for all patients, and patients with limited health literacy may not experience the benefits from these health technologies. Patients with limited health literacy find it difficult to find, comprehend, or apply health information; they are not able to take responsibility for their health and find communication difficult [6]. The aforementioned digitalization of care requires even more skills of patients, such as the ability to operate devices and navigate on the web, understand and use health apps, and apply digitalized nursing consultations. These skills are called digital health literacy or eHealth literacy [7]. Digital health literacy is not only a technical skill set but the ability to engage with digital technologies in effective, safe, and helpful ways to achieve health goals [8]. It is influenced by sociodemographic factors, such as age, educational level, income, perception of the internet as a health source, and social support [9-11]. In the Dutch context, these inequalities are substantial: it is estimated that 1 in 3 people have a limited health literacy [12], and approximately 20% experience difficulties with digital skills [13]. It can be expected that, in a hospital setting, this number may even be higher because patients may experience tension. For example, in acute or critical care settings, patients may be sedated or intubated, limiting direct interaction, and nurses often need to communicate primarily with family members rather than the patients themselves.

Given these challenges, nurses could play an important role in the care of patients with limited (digital) health literacy, starting with their ability to recognize these patients. However, recognizing these patients is difficult, and studies have shown that nurses often overestimate the health literacy of their patients [14,15]. In addition, limited evidence exists regarding nurses’ knowledge and awareness of patients’ (digital) health literacy, and little is known about the practices nurses currently use to identify patients with limited (digital) health literacy in clinical practice. Once patients with limited (digital) health literacy have been identified, nurses need practices to support them. Although previous qualitative research has examined how nurses assess and support patients with limited health literacy, attention to the digital component is mostly lacking. In addition, concept descriptions of (digital) health literacy are available in the literature, and evaluations of nursing practices or interventions remain scarce. Given the increased use of health technologies, an understanding of recognizing and subsequently supporting patients with limited (digital) health literacy by nurses in hospitals is needed.

In conclusion, the increased availability of health technologies is likely to have changed nurse-patient communication. Yet, to date, only a few studies have examined how the rapid digitalization of health care specifically reshapes the 6 communication functions between nurses and patients in hospital settings. In addition, little is known about how nurses can recognize or support patients who experience difficulties in using these health technologies. Therefore, in this qualitative study, we aim (1) to explore which health technologies are used by nurses within a hospital context and how the 6 functions of nurse-patient communication have changed through the use of these technologies and (2) to examine nurses’ current practices, as well as the barriers they experience, in recognizing and supporting patients with limited (digital) health literacy.

## Methods

### Design and Setting

A qualitative descriptive study was conducted, using thematic analysis of semistructured interviews. The interviews were conducted in Isala, a teaching hospital in Zwolle, and at Windesheim University of Applied Sciences in Zwolle, and a vocational education program at Drenthe College in Meppel, all in the Netherlands. This study is reported in concordance with the COREQ (Consolidated Criteria for Reporting Qualitative Research) guidelines [16].

### Participants

Purposive sampling was used to obtain a heterogeneous sample of nurses, intentionally selecting participants with diverse specialisms and experiences. Inclusion criteria were current employment at Isala, a teaching hospital in Zwolle, the Netherlands, either as a registered nurse or a nurse in training; a minimum of 1 year of clinical experience or completion of at least 1 hospital-based internship; and currently using at least 1 health technology.

### Procedures

Potential participants (17 registered nurses and 6 nurses in training) were invited via email, either directly by the researchers or through their supervisor about the study’s aim, interview topics, and the implications of participation. Of those invited, 2 did not respond or were unable to participate due to time constraints, while the remaining individuals agreed to participate.

Interviews were conducted a few weeks after the invitations were sent, either face-to-face or via video call. Before each interview, participants provided written informed consent.

Interviews took place between November 2022 and January 2023 at the hospital and were conducted by 2 researchers (EMD and JW), both trained in qualitative research. One researcher was employed as a nurse researcher at the hospital, and the other was a lecturer at the University of Applied Sciences. A pilot interview was conducted to assess the clarity and structure of the interview guide, leading to minor adjustments in question sequencing.

A total of 21 interviews were conducted, with 17 held in person and 4 online. All interviews were audio-recorded with participants’ permission and transcribed verbatim. During most interviews, a student assistant was present to help manage time and ensure all questions from the interview guide were addressed. Interviews lasted between 25 and 54 minutes.

### Interview Guide

The interviews were conducted using an interview guide, including 3 main topics.

#### The Use of Health Technologies and Their Impact on the 6 Functions of Patient-Nurse Communication

The data were gathered using the 6-function model of communication (Table 1) [2].

First, nurses were asked how each function was part of the nurses’ work and if any health technologies were used in performing this function. Health technology was defined as the application of organized knowledge and skills in the form of devices, medicines, vaccines, procedures, and systems developed to solve a health problem and improve quality of life [17]. An infographic with examples of health technologies (such as video calling, patient portals, and digital leaflets) used in nursing care was presented to stimulate discussions about their own experiences with these health technologies and experiences with other technologies they may have used. Furthermore, nurses were asked if and how these technologies have changed the specific function. Each participant was interviewed about at least 2 functions. If there was enough time, nurses were asked about more than 2 functions. These 2 functions were rotated between interviews so that all functions were addressed equally.

**Table 1.** The 6-function model of communication [2].

Six functions	Explanation
Fostering the relationship	Emphasizes the importance of mutual trust, understanding, and commitment, as well as agreement about each other’s roles and expectations
Gathering information	Aims to ascertain a correct interpretation of symptoms and establish an adequate nursing diagnosis
Providing information	Aims to provide understandable, accurate, sufficient, and timely information in a manner appropriate to the patient
Decision-making	Aims to make sure decisions are being based on the information and preferences of patients
Enabling disease and treatment-related behavior	Aims to support patients in treatment adherence and a healthy lifestyle
Responding to emotions	Requires nurses to elicit patients’ emotional distress, communicate an understanding of the patient’s emotions, and respond with empathy and support

#### Current Practices in Recognizing and Supporting Patients With Limited (Digital) Health Literacy

The critical incident technique [18] was used to gain insight into current practices in recognizing and supporting patients with limited (digital) health literacy. Nurses were asked to describe a situation where the communication with a patient with limited (digital) health literacy failed, followed by several follow-up questions aimed at elaborating on the situation (eg, “What happened?,” “What went wrong?,” and “What did you do to fix it?”). Then, the opposite was asked to describe a situation wherein the communication was very effective. Again, nurses were encouraged with follow-up questions to elaborate on the situation. Furthermore, some additional questions were asked to gain insight into their current practices regarding recognition, such as, “Can you assess whether a patient you are seeing for the first time can work with that technology?,” and support, such as, “How do you ensure that a patient with limited digital health literacy can work with this technology?”

#### Barriers to Recognizing and Supporting Patients With Limited (Digital) Health Literacy

Nurses were asked which barriers they experienced in recognizing and supporting patients with limited (digital) health literacy. For example, the following questions were asked: Which barriers do you experience in recognizing patients with limited digital health literacy? And how is this for supporting patients with limited digital health literacy?

### Data Analysis

The data were analyzed using a thematic analysis [19] using Atlas.ti 22. Two researchers (EMD, JW) first independently read all transcripts to familiarize themselves with the data and identified relevant text fragments. Coding was conducted independently, and coders met after 3, 6, and 12 interviews to discuss their findings and refine the coding scheme. Any discrepancies were resolved through discussion, and a third researcher (CHCD) was consulted when necessary to reach consensus. After each round of discussion, the coding scheme was further refined. No formal intercoder reliability metrics were calculated, as this is not standard for thematic analysis, in which the focus is more on finding patterns of meaning rather than on numerical agreement. We combined deductive and inductive approaches: deductive coding was guided by the main research questions, while inductive coding allowed subcategories to emerge from the data. Text fragments were first sorted (deductively) into categories in line with the research questions: changes in the 6 functions of communication, practices to recognize limited (digital) health literacy, practices to support patients with limited (digital) health literacy, and experienced barriers in recognizing and supporting patients with limited (digital) health literacy. Within each category, subcategories were developed inductively, allowing themes to emerge from the data.

Data saturation was assessed following Hennink et al [20], who distinguish between code saturation, the point at which no new codes are identified, and meaning saturation, the point at which a richly textured understanding of the themes is achieved. In our study, no new codes or themes emerged after 16 interviews, indicating that code saturation had been reached. A total of 21 interviews were conducted, ensuring that all research questions were comprehensively addressed and meaning saturation was also achieved.

### Ethical Considerations

The study was conducted following the ethical principles of the Declaration of Helsinki. The Medical Research Ethics Committees confirmed that the study (reference number 20221009) is not subject to the Medical Research Involving Human Subjects Act (WMO). Participants provided written informed consent prior to the interview. Participants were able to decline to answer any questions and withdraw from the study at any time up to the time of data analysis. All participant-related data were deidentified and kept by the researcher. Only research team members had access to the data. No compensation was provided, apart from reimbursement for the time participants spent participating in the interview.

## Results

### Characteristics of Participants

The characteristics of the participants (n=21) are presented in Table 2. One nurse worked at the virtual monitoring center, where patients at home are monitored remotely. A chief nursing information officer and 2 nurse information officers also participated; these roles aim to support the integration of health technologies into nursing practice. Additional specialized roles included an electronic medical record (EMR) key user, who acts as a mediator between EMR developers and clinical staff, and a digital health technology coach (“digicoach”) who supports nurses in using digital tools. No compensation was provided, apart from reimbursement for the time participants spent participating in the interview.

**Table 2.** Characteristics of participating nurses (n=21).

Characteristics	Participants, n
Age (y)	
20‐29	10
30‐39	5
40‐49	5
≥50	1
Sex	
Male	4
Female	17
Working in	
Clinical wards	
Abdominal and vascular	2
Urology	1
Oncology	1
Dialysis	1
Intensive care	2
Cardiology	2
Nurses in training across various clinical wards	6
Outpatient specializations	
Pulmonary	1
Wound	1
Oncology	1
Ostomy	1
Virtual monitoring center	1
Other	
Chief nursing information officer	1

### Health Technologies in Patient-Nurse Communication

Nurses indicated using various health technologies, both in the hospital and in the home environment. For example, at the hospital, nurses are using the EMR to report about the patient’s condition, use videos to support their education for patients, and use a projector with images (eg, nature, animals, or cities) to calm patients. The mentioned health technologies were divided into 5 categories: e-interaction, e-monitoring, e-education, e-recording, and e-support (Table 3).

**Table 3.** Overview of health technologies used by the hospital nurses.

Category of health technologies	Health technology
E-interaction	Video consultation, e-coach, translator, email contact (including sending photos), phone call, and notifications for nurses via the app
E-monitoring	Distant monitoring of measurement directly transferred to EMR^a^, online questionnaire about self-reported symptoms, monitoring exercise behavior, and camera surveillance
E-education	Images, digital leaflets, videos, webinars, and websites
E-recording	Electronic medical record, patient portal, and digital diary for family
E-support	Projector with calming images, virtual reality, music, online church services, and VR^b^ cycling: stationary bike combined with a screen

a EMR: electronic medical record.

b VR: virtual reality.

### Health Technology Influence on Patient-Nurse Communication

#### Overview

We analyzed nurses’ responses regarding whether and how health technologies have affected the 6 functions of communication. According to the nurses, health technologies influenced all 6 functions of nurse-patient communication. They reported positive effects on gathering information, providing information, supporting disease and treatment management, and responding to patients’ emotions. In contrast, technology was perceived as making the development of the nurse-patient relationship more challenging, and it was rarely used to support shared decision-making. The results are summarized in Table 4, with + indicating positive changes, – negative changes, and ± changes that were neither clearly positive nor negative, reflecting neutral effect.

**Table 4.** How health technologies have positive, negative, or neutral changed the 6 functions of communication.

Functions	Changes	
Fostering the relationship	Increases patients’ sense of safety (eg, through camera surveillance) (+^a^) Lack of eye contact or nonverbal communication (±^b^) Creates more distance between patient and caregiver (eg, video consultations) (–^c^) Requires more time to build a relationship (–) Patients feel less listened to (–) Less privacy for a patient (eg, through camera surveillance) (–)	
Gathering information	Data are more structured in EMR^d^ (+) Allows measurements to be recorded in the EMR with the patient present (+) Increases safety through automatic transfer of vital signs into the EMR (+) Quick and complete data collection via online questionnaires before or during admission (+) Continuous data collection at home (+) Reduces number of hospital visits through video consultations (+) More insight into the home environment (+) Email consultations offer the opportunity to respond whenever suitable (+) Enables quick and adequate treatment in the home situation through, eg, sending photos of ostomy (+) Missing “the clinical view” (±)	
Providing information	Increases patient insight into medical data through, for example, portal (+) Provides relatives insight into patient well-being via “digital diary” (+) Increases options for multimodal education, which enhance patient understanding (eg, videos) (+) Can be sent in advance to enhance patient preparation (eg, digital leaflets) (+) Check patient understanding in e-education (±) Check patient understanding in e-education (±) Some images are too complex and reduce clarity (–)	
Decision-making	Access to (personal) data can contribute to informed decision-making (+) Retrieving information from EMR is simplified (+) Online decision aid can contribute to shared decision-making (+)	
Enabling disease and treatment-related behavior	Exercise options to stimulate the patient to healthy behavior (+) Requires patients to take a more active role through remote monitoring (±)	
Responding to emotions	Can contribute to distraction, for example, through VR^e^ (which can reduce pain) (+) Enables emotional expression via iPad emoticons (+) Builds reassurance through better understanding of condition via e-education (+) Enables nurses to inform their colleagues about patients’ emotions fostering coordinated care (+) Enhanced comfort in home situation (+) Emotional support after admission through, for example, messages (+) Requires attentive listening to the emotions expressed, as visual cues are not available (±) Technology can create disruptions, for example, nurse paging systems (–)	

a +: positive change.

b ±: changes that were neither clearly positive nor negative.

c –: negative change

d EMR: electronic medical record.

e VR: virtual reality.

#### Fostering the Relationship

Building trust and fostering the relationship between nurse and patient constitutes the basis for successful nurse-patient communication. According to participants, this function has changed considerably with the increased use of health technologies, such as video consultation and notifications via patient portals and apps, and mostly in a negative way. It causes a lack of eye contact and nonverbal communication, creates more distance between patients and nurses, and requires more time to build a relationship. Some nurses mentioned that their patients feel less listened to because the nurse is typing during the conversation to add results into the EMR. Regarding e-monitoring, nurses thought that this would increase the patient’s sense of safety. However, a negative consequence was also noted, namely that (camera) surveillance could compromise privacy. Finally, the nurses indicated that a good relationship could be established if it starts with a face-to-face interaction before the technology is being used, as is illustrated in the following quote:

*It also does bring a bit of distance but I think you can reduce that distance if you have created trust during a [face-to-face] consultation at the outpatient clinic. And, if you have taken time for that*.

[R10]

#### Gathering Information

The second function of nurse-patient interaction is gathering information: nurses need to gather information about symptoms and health status to reach an adequate diagnosis and treatment (care) plan. According to the nurses, this function has mostly positively changed with the availability of health technology. For example, e-recording provides more structured data of patients and allows a quick search for information, and the possibility to report the provided information into the EMR in the presence of the patient reduces the chances of mistakes. Automatic measurement and transfer of vital signals into the EMR increases safety and reduces workload for nurses. The use of online questionnaires allows for quick and complete gathering of data before or during hospital admission and continuous data collection also at home. The positive changes of e-interaction (eg, video consultations) included more insight into the home environment of the patient and that patients have to come less often to the hospital. In addition, nurses indicated that online consultations are less often interrupted, and email consultations or messages offer the opportunity to respond whenever suitable. For patients with ostomy or wounds, especially, the opportunity to send photos enables quick and adequate treatment in the home situation.

As a downside, nurses emphasized that by e-interaction, they sometimes miss “the clinical view.” This makes listening skills particularly important, as is outlined in this quote:

*It is very different to have a clinical view over the phone or email than live, where you can also watch someone. Yes, you have to listen very carefully and you have to start asking a lot more questions, so you don’t just look at how someone is sitting, but you also ask, what did you just do when someone is out of breath? Because I didn’t see all that. Where are you right now? Are you standing? So I have to start asking very specific questions about a measurement.*

[R8]

#### Providing Information

The third function of nurse-patient interaction is information provision: nurses have to provide clear information tailored to the patient, for example, information about symptoms, diagnosis, disease, and treatment. Nurses felt that this function was mostly changed in a positive way. Regarding e-recording, the patient portal provides more insight for patients into their personal medical data, and a “digital diary on the ward” provides patients and their relatives insight into nurses’ interpretation of patients’ well-being and daily activities. Regarding e-education, websites, visualizations, and videos provide options for multimodal education and thereby enhance patients’ understanding, as is illustrated in the following quotation:

*A video with illustrations is a much better tool and easier than a leaflet, I think. Many leaflets that I hand out, turn out to be still unread at the bedside table by the end of the admission*.

[R5]

Moreover, education via the internet can easily be sent in advance, enhancing patient preparation and improving personalized communication. On the other hand, one nurse indicated that some images are so difficult that they make the information less understandable. When oral education is replaced by e-education, for example, digital leaflets, it is important to check if the information is found and understood by the patient.

#### Decision-Making

Nurses play a pivotal role in facilitating shared decision-making by providing information and taking into account preferences. Currently, nurses use a limited number of health technologies in the decision-making process. Regarding e-recording, patient access to (personal) data can contribute to informed decision-making, and retrieving information by nurses from EMR is simplified. E-support, such as online decision aids, can contribute to shared decision-making. Although most interviewed nurses had not yet incorporated online patient decision aids into their daily practice, these technologies were recognized as potentially valuable additions for shared decision-making with patients. This potential valuable was described by one of the nurses as follows:

*They exist [Patient decision aids], but are not used very often in practice. I was introduced to a digital colorectal cancer decision aid, explaining types of cancer and the advantages and disadvantages of different treatments, help patients choose what they want. I liked that, but in practice, it is hardly used*.

[R11]

#### Enabling Disease and Treatment-Related Behavior

The fifth function includes enabling disease and treatment-related behavior, which involves supporting patients in treatment adherence and promoting a healthy lifestyle. Nurses described different changes in this function resulting from the increased use of health technologies. About e-support, the implementation of virtual cycling increases exercise options to stimulate the patient to healthy behavior.

Regarding e-interaction, the use of health technologies, such as apps and sending photos by email, facilitates a more active role of patients as they are required to make choices and take actions on their own. For example, patients have to make medication intake changes or have to make changes in the wound care treatment, such as stopping flushing the wound. This more active role is mostly regarded as positive but also requires extra effort and skills of the patient, as becomes clear from the following quote:

*I always say [to patients]; care at a distance is good care, but it is care in which you are in charge, you have to take the steps yourself. But the patient also needs to understand those steps*.

[R10]

#### Responding to Emotions

Patients often experience a range of emotions, such as anxiety, anger, and sadness, as a result of their disease. Nurses can play a crucial role in identifying and responding to these emotions, which can provide patients with a sense of support, but is also a basis for other functions, such as adequate information exchange, decision-making, and self-management. Nurses mentioned that health technologies mainly caused positive changes in this function. Technologies mentioned under e-support included music, virtual reality, video calling with relatives, and online church services that can provide distraction and support to patients. For example, one nurse explained how projected images could distract and calm patients:

*For example, you find yourself in a forest that was really an experience, and you notice that patients are really happy with that, so in that way, yes, offering something with images and sound, brings distraction. Being in another world for a moment*.

[R2; an image projector was used]

In a similar way, virtual reality can lower the experienced pain, for example, during wound treatment.

In addition, if patients are not able to talk, an app was used that allows them to digitally indicate emotions by selecting an emoticon on an iPad. This technology makes it possible to express emotions without using voice. E-education can establish reassurance because a better understanding of their condition or treatment can reduce emotional stress. Using e-interaction can contribute to enhanced comfort in the home situation and create the opportunity for emotional support after an admission. Additionally, as a result of new opportunities to communicate digitally with patients (such as via messages, phone consultations, and emails), nurses are required to attentively listen to the emotions expressed by patients as visual cues are not available. Finally, e-recording, such as the EMR, enables nurses to inform their colleagues about patients’ emotions and feelings, fostering comprehensive and coordinated care. On a downside, nurses also admitted that interruptions caused by health technologies, such as nurse paging systems, can affect the emotional support provided by nurses.

### Recognizing Patients With Limited Digital Health Literacy

In the interviews, nurses describe different practices to recognize a patient with limited digital health literacy, which could be divided into three categories: (1) intuition, (2) observation of verbal and nonverbal signals, and (3) explicitly ask and register in EMR (Table 5).

**Table 5.** Current practices in recognizing patients with limited (digital) health literacy.

Current practices and subthemes	Examples
Intuition	
Intuition	Gut feeling, intuition
Observe verbal and nonverbal signals	
Patient characteristics	Advanced age Low educational level Poor lifestyle Comorbidities
Depending on network	Social network Care network
Behavior	Non-compliant with advice Not having a critical attitude No motivation or not taking initiative Asking less or irrelevant questions Use of language: choice of words, writing style Not understanding or not reading health information Only using a few health technologies or difficulties with using them Not willing to use health technologies Excuses or avoiding information or do not want any information
Explicitly ask and register in EMR^a^	
Ask questions to identify limited digital health literacy	Explicitly ask about digital skills and health literacy Apply a validated assessment tool
Ask patients to demonstrate how they use health technologies	Instructions are not carried out sufficiently Ask the patient to demonstrate and observe difficulties
Register in the EMR	Low literacy registered

a EMR: electronic medical record.

#### Intuition

One frequently used answer to the question on how nurses identify patients with limited (digital) health literacy was intuition. Twelve nurses mentioned that they use their gut feeling to assess if patients have limited or adequate (digital) health literacy. It was difficult for them to explain what this was based on, as is illustrated in the following quote: “You have a gut feeling that the patient is going to perform it properly, but on what do I base that ... I don’t know” (R3). Several nurses acknowledged that this nursing practice is quite uncertain and can lead to incorrect assessments.

#### Observe Verbal and Nonverbal Signals

Other nurses were more explicit about the cues they used for inferring limited literacy or digital literacy in their patients. Certain patient characteristics were mentioned, including advanced age, low educational level, multiple comorbidities, and an unhealthy lifestyle. Some of these characteristics were observed, such as being overweight, advanced age, smoking, and shabby clothing. Other characteristics were indirectly deduced, such as a low educational level and the presence of comorbidities.

Nurses also identify patients with limited digital health literacy based on the level of dependence on their social or care network. They either asked or observed during a hospital admission the extent to which patients rely on their family or acquaintances, for example, with reading or understanding health-related information or completing online questionnaires. Hospital nurses can collaborate with home care nurses or health care professionals from other departments to gather additional information of the patient.

Another nursing practice to identify patients with limited (digital) health literacy is by observing certain behaviors or verbal cues, such as lack of critical attitude, noncompliance with advice, asking irrelevant questions, and not being motivated. The following quote explains how a lack of a critical attitude is being used as a cue for limited (digital) health literacy:

*I observe how critical a patient is, or whether he will let everything happen to him. For example, do they take their medication willingly, or do they ask me, what is it or what is it for? I take that into account in my assessment*.

[R1]

#### Explicitly Ask and Register in EMR

Finally, nurses sometimes explicitly asked or assessed (digital) health literacy levels with questions or observed the patient while using the technology. An example of a question that was typically used by nurses to identify patients with limited health literacy was as follows: “Do you feel overwhelmed by the abundance of health information?”

Digital skills were mostly identified with straightforward questions about the patients’ use of email, WhatsApp, or social media. Another nursing practice to identify limited digital literacy was to observe how a patient works with health technologies:

*If we have the equipment on the ward to let them do it themselves and then I will observe them: How does the patient handle it, does the patient know how it works, what does someone do with the explanation? Does it then go right at once or does the patient need more frequent explanations? That does say something about the skills*.

[R5]

Formal assessment tools to measure limited (digital) health literacy were never used, and most of the interviewed nurses did not know about the existence of such tools. During the interviews, it also became clear that low literacy is not or only scarcely registered in the EMR. Yet, several nurses indicated that it would be helpful to report about (digital) health literacy in the EMR to facilitate awareness and enable them to tailor care accordingly. Documentation in the EMR would also ensure that other involved health care providers would be informed so that the patient does not have to be repeatedly questioned about these skills.

### Supporting Patients With Limited Digital Health Literacy

In daily practice, nurses use certain practices to support patients with limited (digital) health literacy in nursing care. Four main practices were identified: (1) create trust, (2) tailor communication, (3) provide additional support, and (4) use the patient network (Table 6).

**Table 6.** Current practices in supporting patients with limited (digital) health literacy.

Current practices	Subthemes
Create trust	Create trust among patients in the health professional Support the self-confidence of the patient Normalize having limited (digital) health literacy
Tailor communication	Repeat information Limit the amount of information and use short explanations Plain or easy language Visualize Use additional methods; write information down or use a whiteboard Check if information given to the patient is understood (teach-back) Step-by-step explanation Clear instructions Ask open questions Develop tailored health technologies
Provide additional support	More frequent contact (phone or face to face) Provide a summary of the provided education and instructions Create a place where patients can ask questions about the use of health technologies Offer the nondigital method
Use the patient network	Involving relatives to support the patient in using health technologies Asking relatives to use health technology instead of the patient When various relatives are receiving the information, relatives can also repeat it for the patient If the patient does not understand the explanation, the nurse can use the patient’s network Relatives can help the patients formulate questions If no relative is available, arrange home care Consult and coordinate with other caregivers

#### Create Trust

To support patients with limited (digital) health literacy, 5 nurses indicated that it is important to create trust. This practice included addressing patients’ distrust of health technologies, supporting and increasing patients’ self-confidence, and also creating trust in health professionals. Nurses mentioned that these are important skills to support patients in understanding health information and support patients in using health technologies. It is crucial to normalize having limited (digital) health literacy and inform patients that many patients are experiencing difficulties in understanding and applying (digital) health information. This was explained by one of the nurses as follows:

*Some patients ask me, why are you asking if I can’t read? I always say, there are quite a lot of people in the Netherlands who cannot read at all. And if that is the case, we like to take that into account because then we can also show you information in a different way*.

[R10]

Normalizing having limited (digital) health literacy hopefully contributes to less shame about having limited (digital) health literacy and makes it easier for the patient to ask questions or to indicate if something is not fully understood.

#### Tailor Communication

Different types of practices exist to tailor the communication to patients with limited (digital) health literacy. These include repeating information, using short explanations, using plain language, and visualizing information. Additional methods to support the patient’s understanding of health information are to write down information or draw pictures and use a whiteboard in a hospital patient room. Certain nurses indicated using the teach-back method to ensure the patient’s understanding. This method asks patients to state in their own words what they understood or remembered from the given health information [21]. Even though this method was mentioned, it was unclear whether all nurses performed it correctly, as several nurses subsequently indicated they were only asking if a patient still had any questions.

#### Provide Additional Support

Providing additional support is described as a useful nursing practice by the majority (n=16) of the nurses. An example was frequent telephone contact to establish more guidance and more opportunities to practice with health technology. Another suggestion was to create a support team who can be reached by phone, where patients can ask questions related to the use of health technologies.

In addition, nurses described that face-to-face communication instead of digital communication should remain possible, recognizing that not all patients may be capable of using health technologies even with extra support. Some nurses described that patients who were not willing or able to use health technologies were excluded from using health technologies. This means that these patients do not have the opportunity to participate and experience the positive effects of it. One nurse indicated that patients were never excluded from home dialysis, including the use of health technologies due to insufficient skills. Excluding none of the patients was a conscious decision, as long as the patient was willing to learn and try. Consequently, this requires nurses to invest time, patience, and creativity in guiding these patients.

#### Use the Patients’ Network

The majority (n=15) of nurses reported that patients’ social networks are also utilized to support patients with limited (digital) health literacy. For example, relatives can assist patients in working with health technologies and repeat important educational instructions: “But you can also check if the son, daughter, or grandchildren might know a little more about technology and could offer help” (R18). Besides, the health care network of a patient (eg, home care) can help if there are no relatives available. Consulting other caregivers about the care for the patient can lead to comprehensive care and appropriate support.

### Barriers to Recognize and Support Patients With Limited (Digital) Health Literacy

Several barriers to recognize and support patients with limited (digital) health literacy exist. Regarding the work environment, one frequently mentioned barrier was the experienced workload. The continuous high workload often leaves limited time to identify patients with limited (digital) health literacy and provide tailored support. Moreover, nurses often see patients only for a very short time, which makes it particularly difficult to get to know the patient very well:

*You don’t know the patients when they enter the hospital and just being admitted. You have to get to know them first. That is sometimes difficult*.

[R4]

Related to the patient, the nurse described that numerous patients feel ashamed about having limited (digital) health literacy and try to disguise this. This makes recognition more difficult. Another barrier is the family members who speak consistently on behalf of patients instead of giving the patient the opportunity to talk. This may make it difficult to gather the right information because it may be the opinion of the family rather than that of the patient. The last barrier is nurses’ own lack of knowledge. Nurses indicate that they have little knowledge about recognizing and supporting these patients. As a result, they often rely on intuition.

## Discussion

### Principal Findings

The results of this study show that nurses in hospitals use a wide variety of health technologies, and this has affected the nurse-patient communication significantly, mainly in a positive way. Nurses mentioned intuition, observing verbal and nonverbal signals, and explicitly asking as nursing practices to identify patients with limited digital health literacy. Nursing practices to support patients with limited (digital) health literacy included building trust, tailoring communication, providing additional support, and engaging the patient’s network. Several barriers were identified in recognizing and supporting patients with limited (digital) health literacy, including a high workload, lack of knowledge, and not knowing the patient.

Our results revealed that according to the nurses, health technologies affected all 6 functions of the de Haes and Bensing’s communication model [2]. Positive consequences were seen in gathering and providing health information, enabling disease and treatment management, decision-making, and supporting patients to handle their emotions. These results are in line with previous research reporting benefits such as reduced unnecessary consultations, improved accessibility for patients, and the potential of messaging systems to facilitate nursing care [22. While our study primarily observed positive changes, we also identified certain challenges. These included, for example, verifying patients’ understanding and addressing complex images or content that required additional explanation for patients. Similar challenges have been reported in other qualitative studies, including the additional workload associated with training patients [23]. These studies have further highlighted nurses’ limited digital skills, as well as the rapid pace of technology implementation [24]. Although overall changes were largely positive, it was notable that our study identified predominantly negative changes within the function of fostering the nurse-patient relationship. Similar challenges were reported in a study using focus groups with primary health care nurses exploring their views on eHealth tools for patient self-management [24]. Nurses expressed concerns about losing personal contact, perceiving digitalization as a potential threat to the nurse-patient relationship [24]. To overcome this challenge, nurses in our study recommended always starting with a face-to-face meeting and switching to digital communication only after the relationship is built. The preference for initial face-to-face consultations, as well as for combining digital and in-person consultations, has also been noted previously [25]. Our findings suggest that task-oriented functions of the 6-function model, such as providing health information and supporting disease management, benefit from technology through increased efficiency, accessibility, and structure. In contrast, relational functions, such as fostering the nurse-patient relationship, are harder to support digitally, as they rely on nonverbal cues and trust-building [2].

To the best of our knowledge, this study is the first to examine the impact of health technologies on nurse-patient communication using the 6-function model of communication [2]. Although the 6-function model was not specifically designed for use in the nurse-patient communication context, it proved to be highly usable, and nurses could easily relate to the 6 identified functions. This is in line with a previous study in which the model was used to examine nurse-patient communication [26]. Although the model was also not developed to study the impact of health technologies, it turned out to be helpful in providing insight into their impact on the various functions of communication.

In nurse-patient communication, it is important to identify the patients who experience difficulties in using health technologies. Patients with limited health literacy are less likely to use health technologies or perceive them as difficult and useless [27], while these patients can actually benefit from them, for example, from the visualization of health education through videos. To reduce exclusion of patients with limited (digital) health literacy, nurses can play a pivotal role in recognizing and supporting these patients. Nurses in our study mentioned different practices to recognize patients with limited (digital) health literacy. These are intuition, observed verbal and nonverbal signals, and explicitly asking patients about their (digital) health literacy skills or asking them to demonstrate the use of the health technology. Previous studies also showed that nurses often use their intuition to estimate health literacy [28] and use verbal and nonverbal cues, such as patients’ use of simple language, facial expressions, or level of interaction [29]. Unfortunately, “intuition” and “observing verbal and nonverbal signals” are very subjective. Relying only on intuitions may not be an appropriate practice, since it is known that nurses frequently overestimate the health literacy of their patients [30]. Some verbal and nonverbal signals, such as lower education, low self-management, less motivation, and low self-efficacy, are indeed associated with limited (digital) health literacy [31,32], but this does not apply to every individual patient and may be difficult to observe. Moreover, for some nonverbal signals, such as looking slobby, evidence is lacking. A potentially more reliable nursing practice is to explicitly ask patients about their (digital) health literacy skills or to demonstrate how they use technology. Although the importance of this was acknowledged by a few of the interviewed nurses, it was not common practice. A reason for being hesitant to ask patients about their health literacy is that many patients feel ashamed about having limited (digital) health literacy [33]. Our results highlight the importance of educating nurses on how to assess patients’ (digital) health literacy in a neutral and nonoffensive manner, so as to avoid causing discomfort or feelings of shame.

Interestingly, our results showed that patients’ levels of (digital) health literacy are only occasionally documented in the EMR, despite the availability of numerous questionnaires to assess digital health literacy [34]. The systematic documentation of this information could reduce the need for repeated assessment and could enhance continuity of care among health care professionals within hospital settings.

To support patients with limited (digital) health literacy, nurses used different practices: building trust, tailoring communication, providing additional support, and engaging the patient’s network. To tailor communication, the “teach-back method,” already proven to be effective [21], was often mentioned. Unfortunately, it appears that the teach-back method is not always performed correctly. A recent review including similar health literacy practices as in our study showed positive effects on patient support, including activation, patient comprehension, and engagement [35]. Strategies often included multimedia or technology-based approaches, simplification of written material, and facilitation of in-person sessions. More research is needed to examine if these practices are actually effective and how nurses can be motivated to use these in daily practice.

Our study revealed that many nurses are experiencing multiple important barriers while recognizing and supporting these patients. These include a high workload, lack of knowledge, and not knowing the patient. For some of these barriers, training could be helpful. Training, encompassing various educational programs and online resources, has been identified as an effective intervention that significantly contributes to improving digital health literacy [36]. Several studies have shown that offering training for nurses on how to recognize and support patients with limited health literacy shows positive effects on knowledge, attitude, confidence, and increased usage of strategies to recognize and support patients with limited health literacy [37-43]. Unfortunately, the digital component is often not included (yet) in these existing training. It is recommended that nursing education and existing training programs systematically integrate the digital component, with particular emphasis on enabling nurses to recognize and support patients with limited (digital) health literacy.

### Strengths and Limitations

A strength of this study is that a heterogeneous group of nurses from different wards and various specializations was included. This contributed to more generalizable results regarding nurses working in hospitals. This study was conducted in the Dutch hospital context, where the implementation of health technologies is relatively advanced. However, it remains unclear how these findings translate to other countries, as differences in health care systems, digital developments, digital literacy of nurses, and organizational structures may influence the adoption and impact of health technologies. Further research in diverse international contexts is needed to explore their applicability beyond the Netherlands.

In this study, we selected participants who had at least some experience with health technologies to explore their perspectives based on actual use. As a result, our findings primarily reflect the experiences of nurses with some engagement in digital technologies. Future research including nurses with little or no prior experience could provide additional insights into potential barriers and challenges.

### Future Research

Since this study primarily used self-reported data, it would be valuable to observe actual nursing behaviors in practice, for example, how frequently nurses use strategies to recognize and support patients with limited digital health literacy. Furthermore, gaining a deeper understanding of patients’ perspectives is equally important. Patients with limited digital health literacy may experience challenges with health technologies that differ from or overlap with those faced by nurses. It is still unclear to what extent patients recognize potential disadvantages or barriers related to technology use. Therefore, future research should explore the specific difficulties patients encounter and the types of support they need to effectively engage with digital health tools.

Besides the patient perspective, future research may explore potential differences across hospital departments, as communication challenges may vary by clinical context. For example, nurses working in intensive care units may face distinct communication challenges, such as needing to communicate primarily with family members or navigating ethical considerations related to digital consent.

Providing training to nurses on how to support patients with limited (digital) health literacy could be beneficial. Such training may help nurses apply structured strategies rather than relying solely on intuition. When developing this training, it is important to cocreate its content with nurses from diverse clinical settings and with varying levels of experience, ensuring that the modules align with current practice. In addition, integrating these competencies into nursing education is essential so that future nurses also develop the necessary skills.

Moreover, involving nurses in the selection and implementation of health technologies is important. Their perspectives can guide the choice and adoption of new technologies, the design of training programs, and the development of user-friendly systems, such as EMRs. This approach helps ensure that digital health innovations are accessible, practical, and responsive to the needs of both nurses and patients. Ultimately, the impact of these technologies depends not only on the tools themselves but also on organizational and systemic factors, including staff readiness, training support, and supportive organizational structures [44-46].

### Conclusions

This study shows that health technologies have strengthened nurse-patient communication and extended de Haes and Bensing’s model [2] to the digital context. Yet, challenges persist, particularly in fostering relationships. It also reveals that systematic (digital) health literacy assessment remains lacking and that current practices to identify and support patients with limited (digital) health literacy may not always be effective. Once effective approaches are established, they should be integrated into nursing care plans and education.

## Abbreviations

COREQ: Consolidated Criteria for Reporting Qualitative Research
EMR: electronic medical record
